# Biomechanical effects of an oblique lumbar interbody fusion combined with posterior augmentation: a finite element analysis

**DOI:** 10.1186/s12891-022-05553-w

**Published:** 2022-06-27

**Authors:** Shengjia Huang, Shaoxiong Min, Suwei Wang, Anmin Jin

**Affiliations:** 1 Department of Spinal Surgery, Orthopedic Center, Zhujiang Hospital, Southern Medical University, Guangzhou, Guangdong 510280 China; 2 Department of Orthopaedics, Zhuhai People’s Hospital (Zhuhai Hospital Affiliated With Jinan University), Zhuhai, Guangdong 519000 China; 3 Department of Spinal Surgery, Peking University Shenzhen Hospital, Shenzhen, Guangdong 518036 China

**Keywords:** Oblique lumbar interbody fusion, Degenerative disc disease, Biomechanical, Posterior pedicle screw and rod, Cortical screw and rod, Finite element analysis

## Abstract

**Background:**

Oblique lateral interbody fusion (OLIF) is widely used to treat lumbar degenerative disc disease. This study aimed to evaluate the biomechanical stability of OLIF, OLIF including posterior pedicle screw and rod (PSR), and OLIF including cortical screw and rod (CSR) instrumentation through finite element analysis.

**Methods:**

A complete L2-L5 finite element model of the lumbar spine was constructed. Surgical models of OLIF, such as stand-alone, OLIF combined with PSR, and OLIF combined with CSR were created in the L3-L4 surgical segments. Range of motion (ROM), end plate stress, and internal fixation peak stress were compared between different models under the same loading conditions.

**Results:**

Compared to the intact model, ROM was reduced in the OLIF model under all loading conditions. The surgical models in order of increasing ROM were PSR, CSR, and stand-alone; however, the difference in ROM between BPS and CSR was less than 0.4° and was not significant under any loading conditions. The stand-alone model had the highest stress on the superior L4 vertebral body endplate under all loading conditions, whereas the end plate stress was relatively low in the BPS and CSR models. The CSR model had the highest internal fixation stress, concentrated primarily at the end of the screw.

**Conclusions:**

OLIF alone significantly reduces ROM but does not provide sufficient stability. Addition of posterior PSR or CSR internal fixation instrumentation to OLIF surgery can significantly improve biomechanical stability of the segment undergoing surgery.

## Background

With the development of minimally invasive spine surgery techniques, oblique lateral interbody fusion (OLIF) has become one of the most widely used techniques for the treatment of lumbar degenerative disc disease in recent years due to the minimal trauma, reduced bleeding, shorter recovery time, and low incidence of neurological complications associated with it [1–3]. Since the size of the intervertebral fusion cage used in OLIF is much larger than that of the conventional posterior fusion cage, and that the fusion cage is placed across the epiphyseal ring of the vertebral body, its biomechanical stability is significantly enhanced [4, 5]. Therefore, OLIF as a stand-alone technique has been used in the management of lumbar spine disorders with some clinical efficacy [6–9]. However, most degenerative disorders of the lumbar spine occur in elderly patients and are affected by many factors, such as the patient’s age, bone condition, intervertebral space management technique, and so on. In addition, postoperative complications such as subsidence and displacement of the fusion cage cannot be overlooked [10]; hence, augmentation with internal fixation instrumentation is required in most cases to improve the stability of fusion [11, 12].

Internal fixation with posterior pedicle screw and rod can result in good biomechanical properties, and can maintain the stability of the spine and promote intervertebral fusion. Thus, it is now typically used for the augmentation of internal fixation of the lumbar spine [13–16]. However, postoperative complications of internal fixation, such as loosening of pedicle screws, rod breakage, and pedicle fracture, have become major limiting factors, especially in elderly patients with osteoporosis [17, 18]. In view of these factors, Santoni et al. proposed the cortical bone trajectory technique in 2009 and applied it to the surgical treatment of spine disorders. This screw placement method maximizes contact with the cortical bone and increases the holding strength of the screw [19]. Overall, the choice of posterior internal fixation technique in OLIF remains controversial. To the best of our knowledge, few studies have investigated the biomechanical properties of OLIF combined with posterior augmentation. This study aimed to use finite element analysis to construct a model of the intact L2-L5 lumbar spine (Intact) and three surgical models: stand-alone OLIF (SA), OLIF combined with pedicle screw and rod fixation (PSR), and OLIF combined with cortical screw and rod fixation (CSR). Further, we compared and analyzed the biomechanical differences in OLIF combined with different posterior internal fixation methods during movements such as anterior flexion, posterior extension, lateral flexion, and rotation, with the aim of providing a reference value for the clinical application of OLIF.

## Methods

### Finite element (FE) model of L2–L5

A healthy adult male volunteer (height: 175 cm, weight: 68 kg) was selected. History of spinal deformity and lumbar disease was ruled out through X-ray imaging. A 64-slice spiral computed tomography (CT, Somatom Sensation 64; Siemens, Germany) at Zhuhai People’s Hospital was used to scan the whole spine of the volunteer with a slice thickness of 0.625 mm. Acquired CT data were imported into Mimics Research 20.0 software (Materialise Inc., Leuven, Belgium) in DICOM format. Region growing, threshold segmentation, manual editing, and other operations were used to capture the bone structure of the L2-L5 lumbar spine, and a basic three-dimensional contour model of the lumbar spine was generated.

The above data were imported into Geomagic Studio 2012 (3D Systems, Inc., Rock Hill, South Carolina, USA) for smoothing, denoising, curve surface construction, and other modifications, and for processing to generate bony contours of the lumbar vertebrae. Next, solid models of cortical bone, cancellous bone, intervertebral disc, cartilage end plate, and articular cartilage were constructed using SolidWorks 2015 computer-aided design software (Dassault Systèmes SolidWorks Corporation, Waltham, Massachusetts, USA), and a three-dimensional geometric model of the lumbar spine was reverse engineered. The thickness of the cortical bone and cartilage end plate was 1 mm, the nucleus pulposus accounted for about 30–40% of the intervertebral disc volume, and the articular cartilage closely approximated the articular surface and was set to a thickness of 0.2 mm [20–23].

**Fig. 1:**
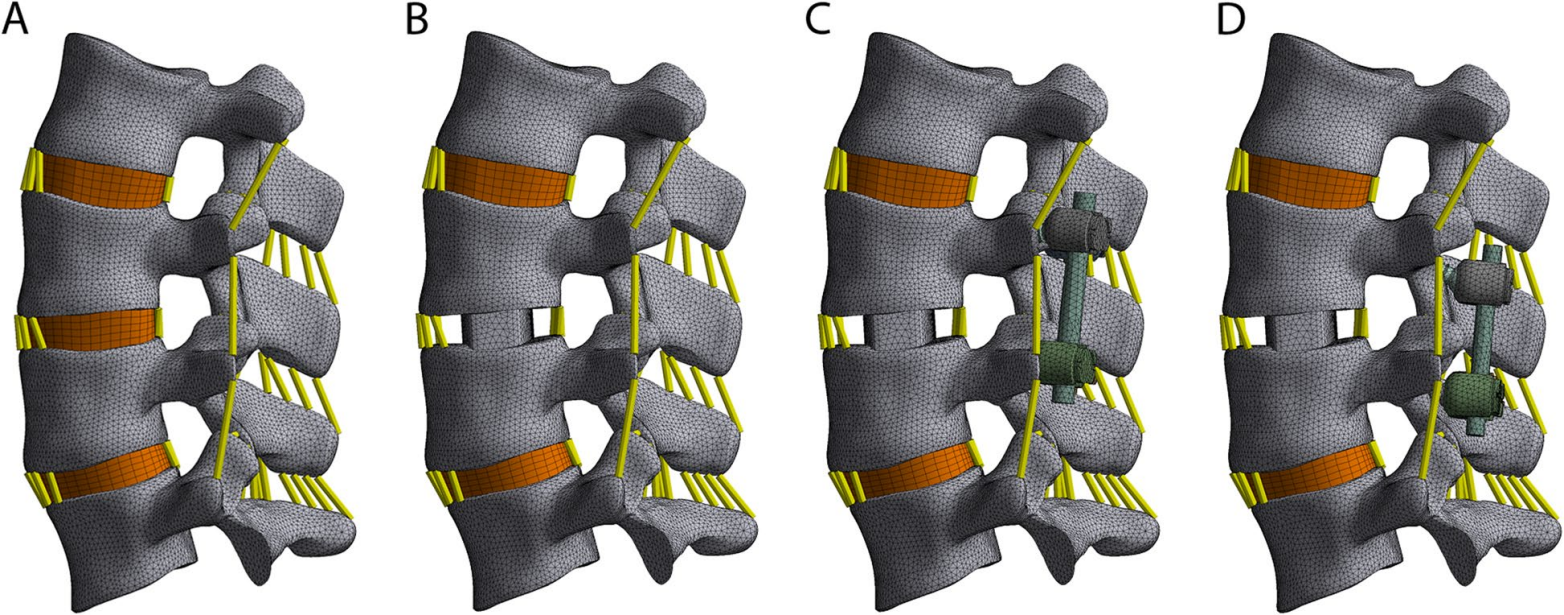
Finite element models in the current study. **a** Finite element (FE) model of the intact L2-L5 spine, **b** FE model of the OLIF stand-alone(SA), **c** FE model of the OLIF combined with pedicle screw and rod (PSR), **d** FE model of the OLIF combined with cortical screw and rod (CSR)

Finally, the constructed solid models were imported into ANSYS Workbench 18.0 software (ANSYS, Ltd., Canonsburg, Pennsylvania, USA) for ligament reconstruction, including anterior longitudinal ligament, posterior longitudinal ligament, ligamentum flavum, capsular ligament, interspinous ligament, supraspinous ligament, and intertransverse ligament reconstruction. The position and structure of all ligaments were accurately simulated as previously described [24]. LINK180 elements were used to simulate the function of the ligaments, and only bear tensile force. Model meshing was conducted using optimal elements, and a high-quality mesh was obtained through mesh convergence analysis to reduce the calculation error. The lumbar spine structure was set as an isotropic linear elastic material, and in the end, a complete three-dimensional FE model of the L2-L5 lumbar spine was constructed by assigning material properties to the model (Fig. 1a). The complete model comprised 638,146 elements and 347,461 nodes.

### Establishment of FE models of OLIF combined with posterior instrumentation

In this study, three-dimensional geometric models of internal fixation instrumentation were constructed based on the actual parameters of screws, connecting rods, and cages, using the part interface of SolidWorks 2015. Specifically, the length and diameter of the pedicle screw were 45 mm and 6.5 mm, respectively; the length and diameter of the cortical screw were 35 mm and 4.5 mm, respectively; the diameter of the connecting rod was 5.5 mm; and the length, width, and height of the intervertebral fusion cage were 50 mm, 18 mm, and 12 mm, respectively. The anterior and posterior sides differed in height, the upper and lower surfaces formed an angle of 6°. For all OLIF models, the “Boolean calculation” is used to remove the part overlapping with the vertebral body, and realized the geometric matching between the fuser and the endplate interface. In order to simplify the influence of sawtooth on the surface of fuser, rectangular surface is used to replace sawtooth surface in modeling. The purpose of this study was to evaluate the biomechanical effects of surgical segments with bone fusion combined with different posterior internal fixation. Therefore, the fusion cage and vertebral endplate, pedicle screw and vertebral body, pedicle screw and connecting rod were regarded as integration. The spinal fixation instrumentation used in this study were the CLYDESDALE spinal system and the CD HORIZON spinal system (Medtronic Sofamor Danek, Memphis, Tennessee, USA).

The study design was based on surgical methods. The L3/4 vertebral segment was chosen as the surgical segment, and surgical resection of the L3–4 cartilage end plate, nucleus pulposus, and part of the annulus fibrosus was simulated. Next, the CLYDESDALE fusion cage was inserted into the L3–4 intervertebral space from the left side, and the interface was fixed, preserving the intact structure of the posterior lumbar spine. Subsequently, SolidWorks 2015 was used to assemble three-dimensional solid models of the L2-L5 segment of the lumbar spine, the fusion cage, and the screw and rod system. Finally, the models of the intact lumbar spine (Intact), stand-alone OLIF (SA), OLIF combined with PSR, and OLIF combined with CSR were constructed (Fig. 1). The fusion cage was fixed in the target intervertebral space, and the same position was used in all surgical models.

The tissue structure and implant material properties in this study were as previously described (Table 1) [23, 25–27]. The articulating surfaces of all joints in the model were defined as surface-surface contact elements with friction coefficients of 0, and all other contact types were set as bonded contacts [23].

**Table 1:** Material properties used in finite element model

Component	Element Type	Young Modulus (MPa)	Poisson Ratio	Cross-Sectional Area (mm^**2**^)
Bone				NA
Cortical bone	Solid186	12,000	0.3	
Cancellous bone	Solid186	100	0.3	
Posterior bony elements	Solid186	3500	0.25	
Intervertebral disc				NA
Annulus fibrosus	Solid187	1	0.499	
Nucleus pulposus	Solid187	4.2	0.45	
Endplate	Solid187	1200	0.29	
Cartilage	Shell163	35	0.4	
Ligaments				
Anterior longitudinal	link180	7.8	0.3	63.7
Posterior longitudinal	link180	10	0.3	20
Ligamentum flavum	link180	15	0.3	40
Capsular	link180	7.5	0.3	30
Interspinous	link180	10	0.3	40
Supraspinous	link180	8	0.3	30
Intertransverse	link180	10	0.3	1.8
Implants				NA
Cage (PEEK)	Solid186	3600	0.3	
Screws and rods (titanium)	Solid186	110,000	0.3	

*NA* Not applicable, *PEEK* Polyetheretherketone

### Boundary and loading conditions

All directions of movement of the lower surface of the L5 vertebral body were constrained and fixed, and a vertical load of 400 N was applied to the upper surface of L2 to simulate the axial load (upright state) of the human body’s weight on the spine. A torque of 7.5 N m was applied in different directions to simulate six different physiological movements of the human body: anterior flexion, posterior extension, left and right lateral flexion, and left and right rotation [28, 29]. The effects of different types of internal fixation instrumentation on the biomechanical stability parameters related to OLIF, including the range of motion (ROM), end plate stress, and internal fixation screw and rod stress, were analyzed and compared.

## Results

### Validation of the model

**Fig. 2:**
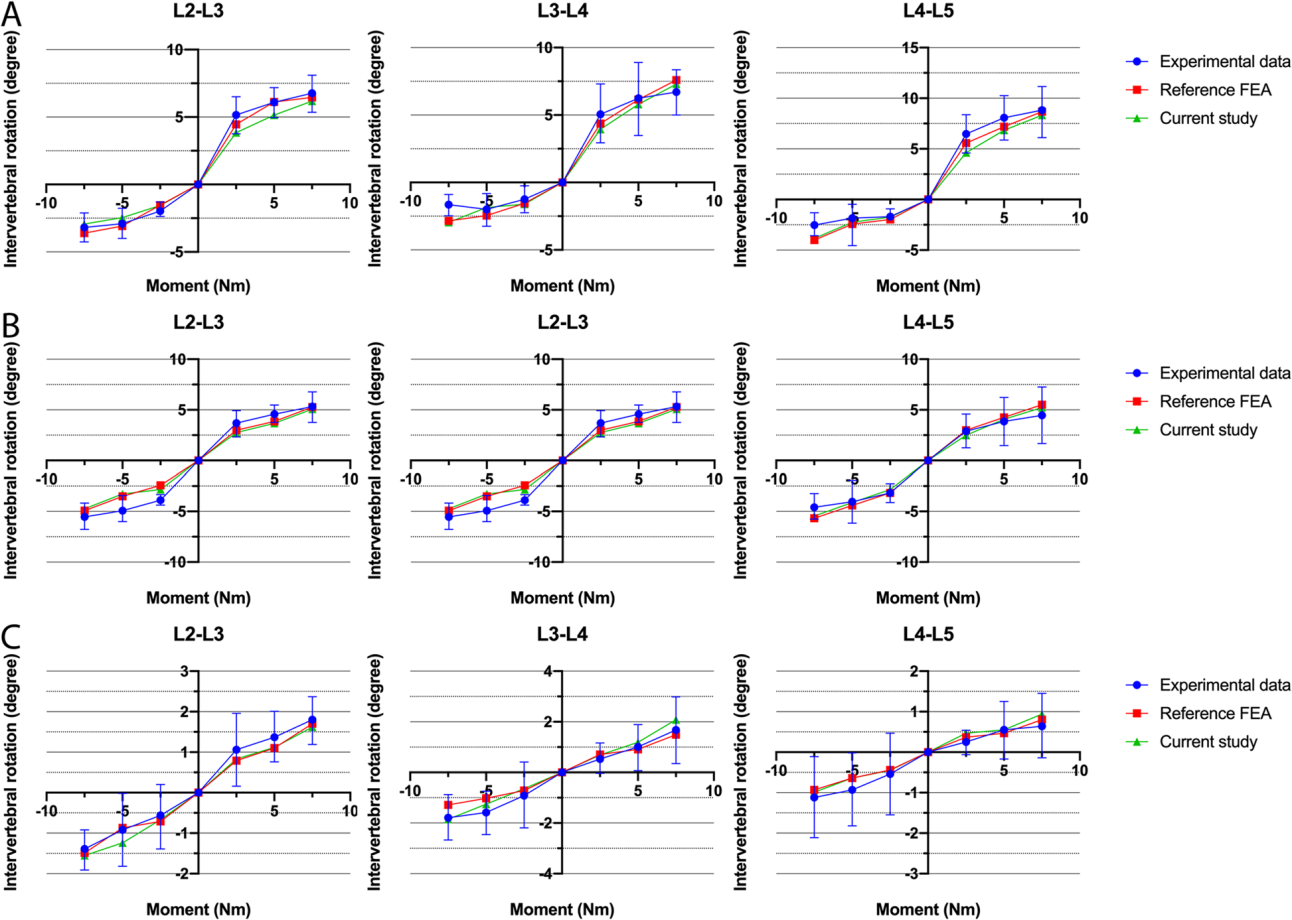
Comparison of the data of three spinal levels. The mean and standard deviation of ROM in three spinal levels (L2-L3, L3-L4, and L4-L5) were obtained by the FE models in this study compared with experimental data (Panjabi et al.1994) and reference FEA data (Ming Xu et al. 2017): **a** under flexion (+) and extension (−), **b** under right (+)/left (−) lateral bending, **c** under right (+)/left (−) axial rotation. Note: the dots represent the mean of ROM and the error bars indicated standard deviation

The effectiveness of the complete L2–L5 finite element model of lumbar spine was verified according to the previous research results of Panjabi et al. [30, 31] Specifically, three different movements — flexion-extension, bilateral axial torque, and bilateral axial bending (2.5 N m, 5 N m, and 7.5 N m) were applied. The ROM experimental results for L2/3, L3/4, and L4/5 were compared with the data from the cadaveric experiment and FE experiment described above (Fig. 2). The results of this study were consistent with data from the literature, proving that the FE model is effective and reliable.

### ROM

**Fig. 3:**
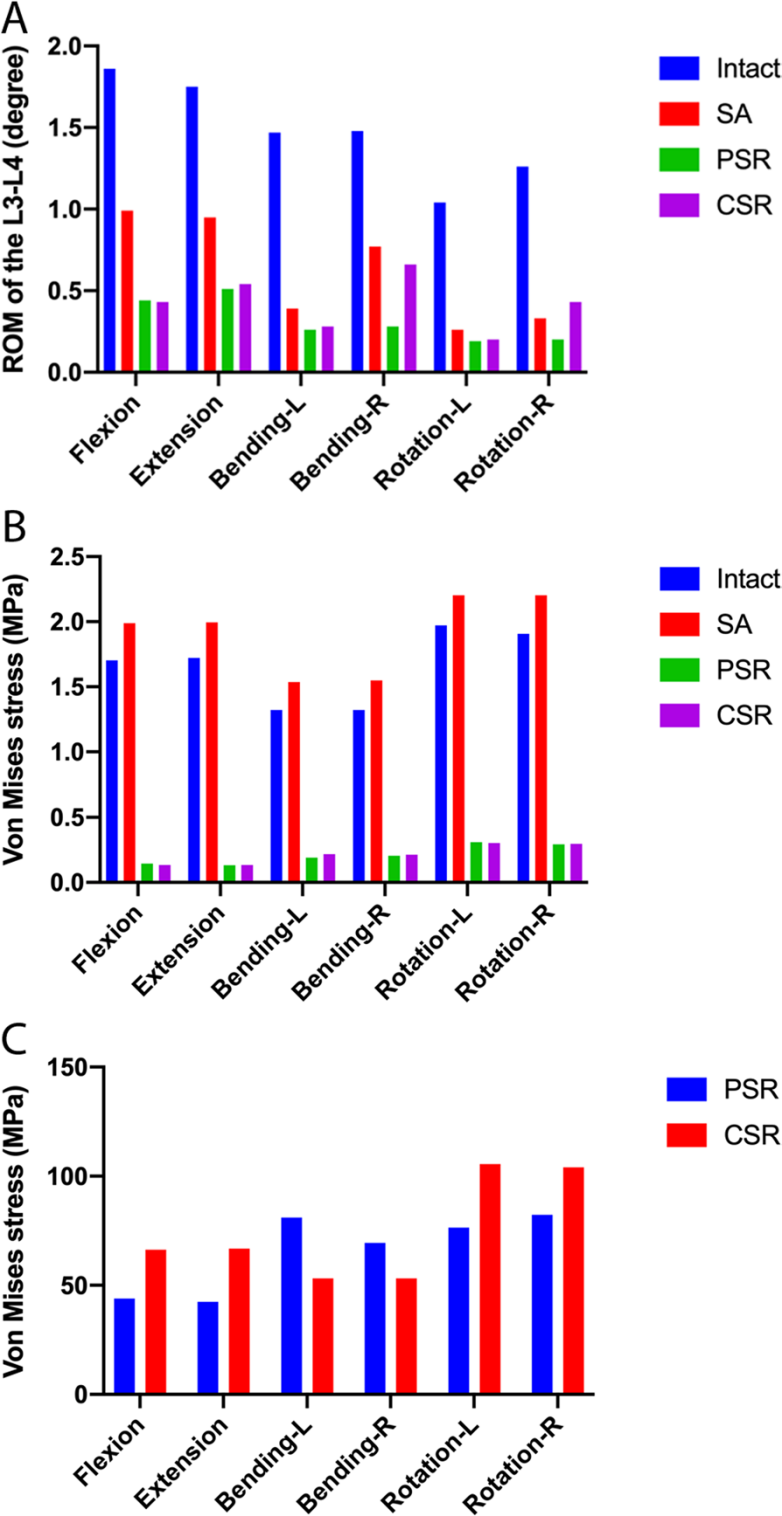
Comparison of experimental results among all models. Comparisons of **a** range of motion, **b** L4 superior endplate stress, and **c** posterior instrumentation stress for the intact model and surgical procedures. ROM, range of motion; Intact, intact model; SA, stand-alone; PSR, pedicle screw and rod; CSR, cortical screw and rod

The ROM of each surgical model under a combined load of 400 N and 7.5 N m is illustrated in Fig. 3a. Compared to the intact FE model, the ROM of the L3-L4 segment was significantly reduced after OLIF in all loading conditions, especially during flexion and rotation. When comparing all the surgical models, the stand-alone OLIF (SA) model showed the largest ROM value under all loading conditions, whereas the OLIF combined with PSR model had the smallest ROM. In addition, the ROM of OLIF combined with CSR model was also significantly lower than that of the SA model, especially during flexion and extension. The difference in ROM between OLIF combined with PSR and OLIF combined with CSR was less than 0.4°, and was not significant under all loading conditions (Fig. 3a).

### Stress of the endplate

**Fig. 4:**
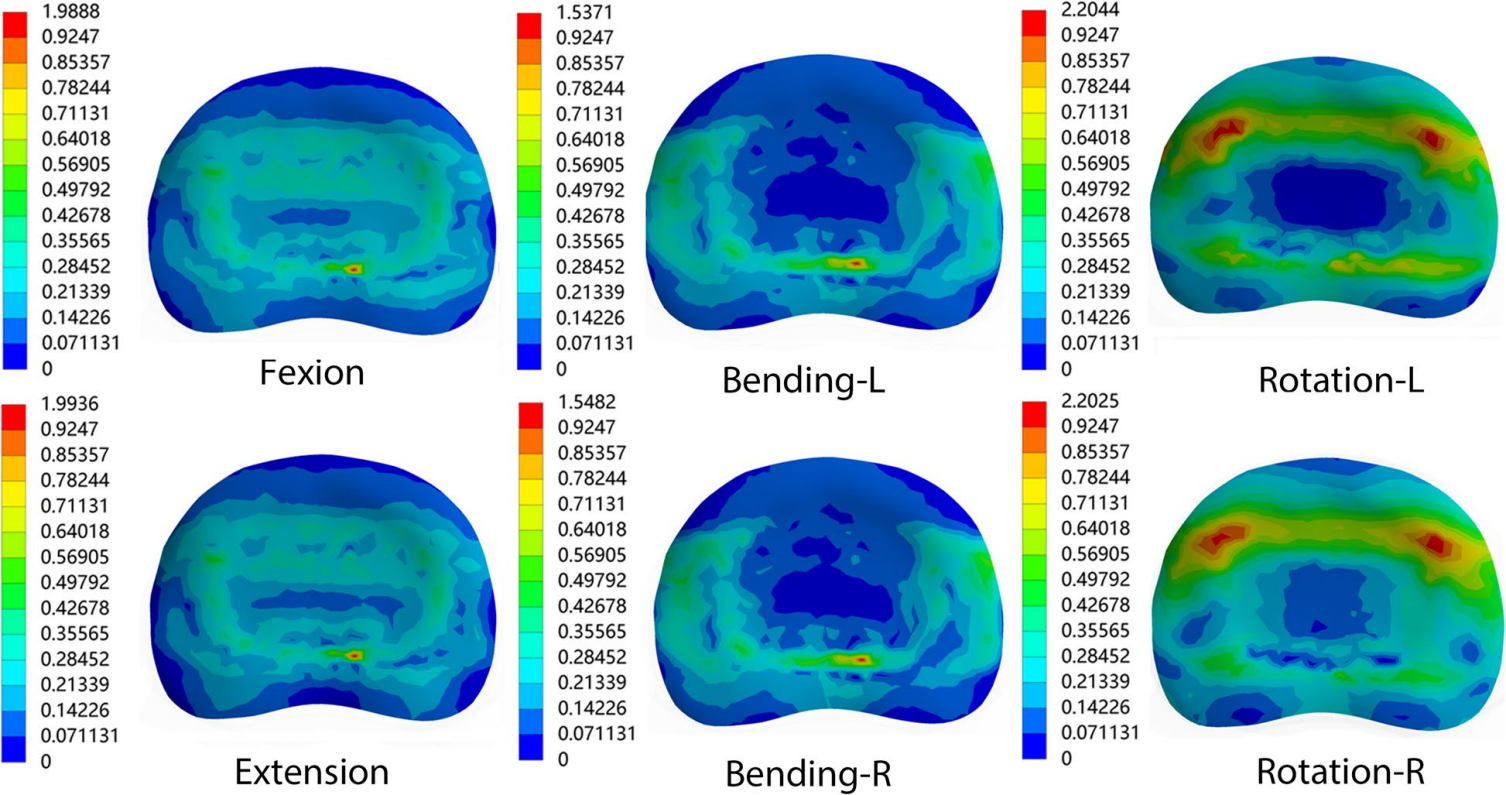
Stress distributions in the L4 superior endplate of OLIF stand-alone (SA) model. L, left; R, right

Under all loading conditions, the maximum stress on the superior end plate of the L4 vertebral body in the SA model was higher than in the other surgical models, but the difference was small compared to the Intact model. When the three different surgical models in the study were compared, the end plate stress in the PSR model was still the lowest in all loading conditions, but there was no significant difference in the actual end plate stress between the PSR and the CSR models under all loading conditions. Figure 3b depicts the peak Von Mises stress on the L4 vertebral body end plate under different directions of motion and in the three different surgical models. The stress color map of the superior L4 vertebral body end plate in the SA model is illustrated in Fig. 4.

### Stress on the internal fixation

**Fig. 5:**
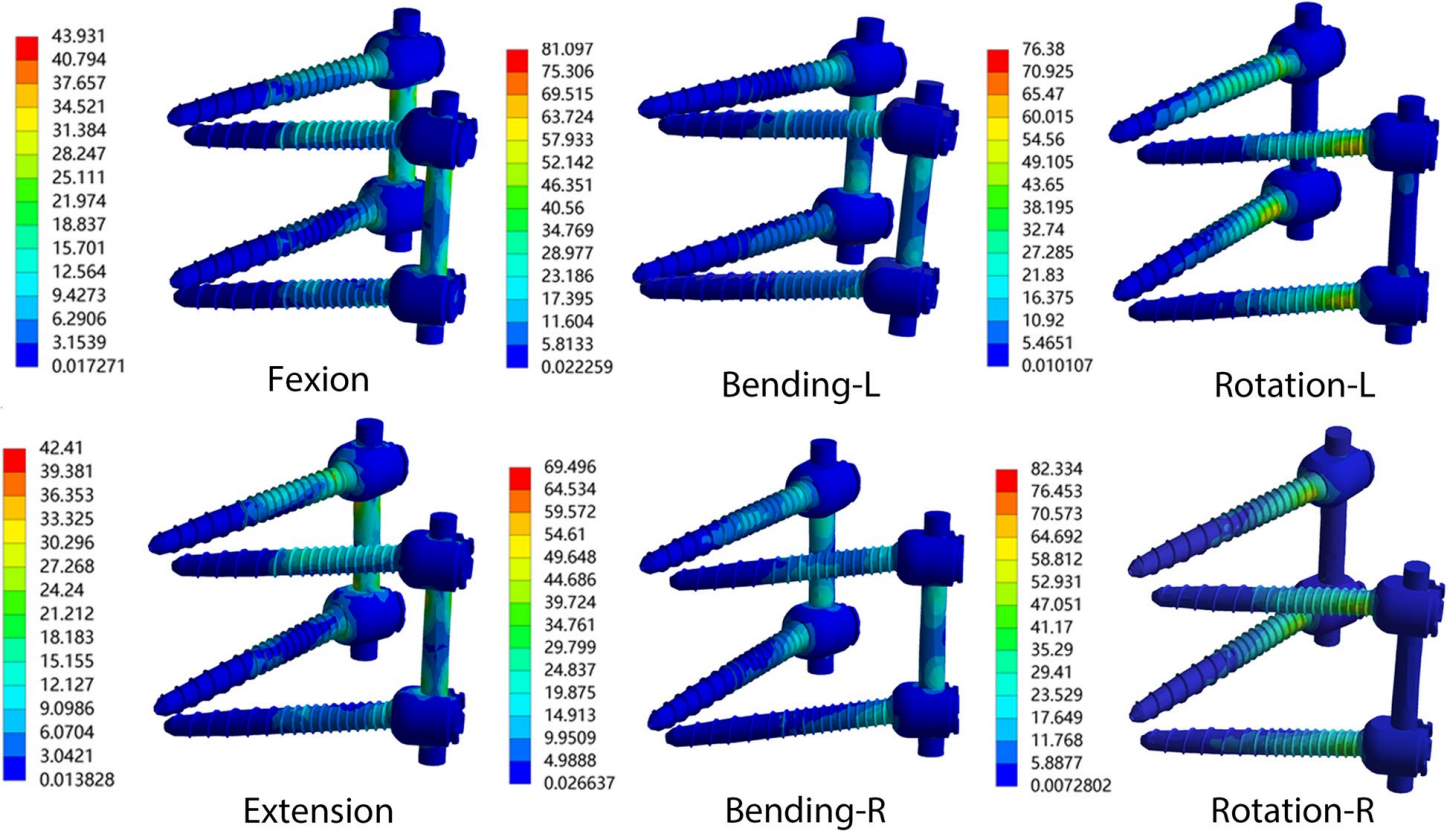
Distributions of instrumentation stress for OLIF combined with pedicle screw and rod (PSR) model. L, left; R, right

In the PSR model, the internal fixation instrumentation bore less stress during flexion, extension, and rotation, and greater during lateral flexion compared with the CSR model. In the CSR model, the stress on the pedicle screw during flexion-extension and rotation was higher than that in the BPS model by 54.2 and 32.0%, respectively. The pedicle screw reached peak stress (105.6 MPa) during rotation in the axial direction in the CSR model. In addition, the maximum stress on the internal fixation instrumentation in each surgical model was found to be concentrated at the end of the pedicle screw (Fig. 5). The peak Von Mises stress of the two surgical models is illustrated in Fig. 3c, and the stress color map of the PSR model is presented in Fig. 5.

## Discussion

Currently, lumbar interbody fusion (LIF) represented by posterior lumbar interbody fusion (PLIF) or transforaminal lumbar interbody fusion (TLIF) remains the most widely used surgical technique. These procedures can directly lead to decompression of the spinal canal, which results in good fusion rates and clinical outcomes [32–34]. However, fusion cages inserted via a posterior approach requires removal of important stabilizing structures in the spine (such as the facet joints), and repeated traction on the dural sac and nerve roots may cause neurological damage [35–37]. In 2012, OLIF was reported for the first time as a technique that could reduce surgical injury and further improve the efficacy of spine fusion [38], avoiding the anatomical problems described above. OLIF involves direct access to the lesioned intervertebral disc via the physiological space between the retroperitoneum abdominal vascular and the psoas major muscle. Therefore, the oblique lateral approach allows safer placement of a fusion cage into the intervertebral space without being limited by the bony structure of the spine, the nerve roots, and the dura mater [3, 39]. In addition, the OLIF technique provides a number of biomechanical advantages. The surgery completely preserves major stabilizing structures such as the anterior longitudinal ligament and the anterior annulus fibrosus, allowing the anterior longitudinal ligament and the posterior ligamentous complex (PLC) to form a coupled motion response [40]. Therefore, the anterior longitudinal ligament provides greater resistance during flexion-extension and axial rotation of the spine [41].

Since the surgical instrumentation of OLIF cannot directly decompress the spinal canal laterally, nor can compressive objects such as herniated intervertebral discs, hypertrophic ligamentum flavum, and hyperplastic facet joints be removed, its clinical efficacy derives primarily from indirect decompression, caused by (1) opening of the intervertebral space with a wide fusion cage, (2) restoring the height of the intervertebral foramen, (3) increasing the tension of the posterior longitudinal ligament, and (4) improving the sagittal sequence of the spine [8, 42]. A prospective retrospective analysis by Tempel et al. [43] involving 297 patients (623 levels) who underwent stand-alone lateral fusion found that fusion cage subsidence was a major predictor of revision surgery following stand-alone lateral fusion. Notably, cage subsidence has become a common complication following stand-alone OLIF. Many studies have indicated that cage subsidence is related to bone density, fusion level, cage position, cage height, and pedicle screw internal fixation [44, 45]. Therefore, prevention of cage subsidence following OLIF and maintenance of postoperative efficacy have received increasing attention. Several researchers have reported the use of OLIF combined with posterior internal fixation instrumentation to maintain stability at the operated level [34, 46, 47]. However, only a few studies have evaluated the biomechanical properties of OLIF combined with posterior internal fixation instrumentation.

The purpose of the present study was to analyze and compare the biomechanical stability of OLIF combined with different posterior internal fixation instrumentation using three-dimensional FE analysis. Standard lumbar posterior internal fixation instrumentation includes PSR and CSR. Previous studies have reported that laterally inserted interbody cages significantly decrease ROM at the operated segment compared to PLIF, TLIF, and anterior LIF [48]. The present study also demonstrated that stand-alone OLIF significantly reduced ROM in all directions of motion compared to the Intact model, indicating that stand-alone OLIF provides some degree of stability at the operated segment.

The CLYDESDALE interbody fusion cage provides immediate stability to the operated segment through “distraction-compression stabilization,” making the stand-alone OLIF technique possible [49, 50]. This is due to the use of a wide fusion cage in OLIF, which not only increases the contact area with the end plate, but the 6° angle between the superior and inferior surfaces helps to restore the height of the intervertebral space and improve the lumbar spine sequence. Clinical trials have also similarly concluded that stand-alone fusion fixation can improve stability of the operated segment in all directions of motion [12]. However, the study also found that the SA model had the highest stress on the superior L4 vertebral body end plate among all surgical models and in all directions of movement, suggesting that the superior end plate may bear the highest reaction force from the fusion cage in stand-alone OLIF, which also significantly increases the risk of cage subsidence. Andrea et al. [51] reported that a wider cage and a more anterior position reduces mobility but also increases the risk of cage subsidence. Therefore, we use OLIF combined with a posterior internal fixation system as a routine surgical procedure in clinical practice. Related biomechanical and clinical studies have demonstrated that posterior instrumentation provides strong fixation to the operated segment, not only sharing most of the load on the superior end plate but also significantly reducing the load on the anterior spinal column [20, 52, 53].

In the traditional sense, laterally inserted intervertebral fusion cages combined with posterior internal fixation instrumentation can significantly improve the biomechanical stability of the operated segment, and not only effectively prevent subsidence and displacement of the fusion cage, but also considerably promote intervertebral fusion. Recently, anterior stand-alone interbody fusion cages have been used in clinical practice, and both their fusion rate on imaging and success rate are acceptable. However, fusion cages inserted through the oblique lateral approach combined with posterior internal fixation instrumentation can result in a stronger and more stable structure [41, 54, 55]. The results of our study indicated that the PSR model not only had the smallest ROM among all surgical models under the same loading conditions, but also had the lowest stress on the L4 superior end plate stress (Fig. 3a, b), suggesting that vertebral pedicle screw fixation in OLIF surgery has significant biomechanical advantages with respect to both ROM of the fixed segment and stress on the end plates of the adjacent vertebral bodies. Consistent with previous reports [56], the present study did not find significant biomechanical differences in the relative mobility and end plate stress in the PSR and CSR structures, indicating that the combination of OLIF with posterior internal fixation instrumentation can significantly limit the mobility of the operated segment, further improving its stability. Posterior internal fixation instrumentation not only reduces stress on the end plate and the cage, but also maintains the indirect decompression effect after OLIF. The PSR and CSR structures in OLIF have good biomechanical stability in all directions of motion, suggesting that both CSR and PSR can be used with OLIF. Alternative or mixed posterior lumbar internal fixation methods can be used if cortical bone screws or pedicle screws cannot be inserted due to technical problems such as anatomical variation or intraoperative complications.

The yield strength of internal fixation with a titanium alloy screw has been previously reported as 795–827 MPa [57]. In the present study, the peak stress on the screw and rod in each surgical model was less than this yield strength. In addition, according to previous research, the fatigue strength of titanium alloy internal fixation is 500 MPa [58]. In this study, we compared the static stress and yield strength of internal fixation after lumbar spine surgery. The results in Fig. 3 show that the maximum stress is less than the yield strength of the internal fixation and far less than the fatigue strength of the titanium alloy, which indirectly proves that the internal fixation method in this study has good fatigue resistance. However, fatigue intensity is a stress limit for multiple repeated loads. Thus, static stress cannot directly represent fatigue strength. It is necessary to test the fatigue strength of internal fixation in vivo in future study. Therefore, the surgical model did not suffer from long-term stress concentration resulting in postoperative fatigue and fracture of the screw and rod, which in turn could affect the clinical efficacy of the procedure. The results of FE analysis revealed that except for lateral flexion, the stress on the screw and rod in the PSR model was lower than that in the CSR model during flexion, extension, and rotation, but the difference between the two was small. This indicates that when OLIF is combined with posterior internal fixation, the biomechanical differences between different types of posterior internal fixation instrumentation are only minor. Therefore, in clinical practice, cortical screws or pedicle screws can be used as an augmented posterior fixation for OLIF. The peak stress of 105.6 MPa on internal fixation with the screw and rod was attained during axial rotation, but this was also far lower than the yield strength. This can be attributed to the fact that the surgical models constructed in the present study were all models of bone graft fusion, and the screw and rod system and the interbody fusion cage each share part of the stress. This also suggests that patients must wear a waistband to restrict waist movement in the early stages after OLIF to prevent postoperative failure of internal fixation. In addition, the internal fixation stress color map reveals that the stress on internal fixation is often concentrated at the end of the screw in both CSR and PSR, (Fig. 5), which is consistent with the sites commonly fractured by screw and rod internal fixation after lumbar spine surgery [59].

### Limitation

FE analysis has many advantages when applied to biomechanical studies of the spine, but it currently cannot be used to construct a full lumbar spine model that includes the paravertebral muscles. As a result, the effects of the surrounding muscles and soft tissues on the biomechanics of the spine have not been evaluated. Second, human tissues are composed of complex, biologically active structures. Material properties were assigned based on the parameters given in the literature, but a gap still exists between these values and those from biomechanics experiments in actual humans. In addition, actual lumbar spine models vary between individuals, and the model in the present study does not account for the degree of degeneration and other individual biological variations. Although the FE model in the present study has been validated in previous studies, it requires further validation by in vitro biomechanical experiments to serve as a true biomechanical simulation.

## Conclusions

Surgeons should consider both the biomechanics of the spine and the individual condition of the patient when selecting the appropriate augmented supplemental fixation technique for OLIF. Posterior lumbar fixation instrumentation provides the most reliable biomechanical stability in OLIF, while stand-alone OLIF does not provide sufficient stability. Second, OLIF combined with different posterior fixation instrumentation (CSR and PSR) exhibits no obvious biomechanical difference in all directions of motion. Therefore, OLIF combined with CSR or PSR structures can provide similar biomechanical stabilization efficacy for fusion and fixation of the operated segment.

## Data Availability

The datasets used and/or analyzed during the current study are available from the corresponding author on reasonable request.

## Abbreviations

OLIF: Oblique lateral interbody fusion
PSR: Posterior pedicle screw and rod
CSR: Cortical screw and rod
FE: Finite element
ROM: Range of motion
SA: Stand-alone
CT: Computed tomography
PLIF: Posterior lumbar interbody fusion
TLIF: Transforaminal lumbar interbody fusion
PLC: Posterior ligamentous complex
